# Community health promotion and medical provision for neonatal health—CHAMPION cluster randomised trial in Nagarkurnool district, Telangana (formerly Andhra Pradesh), India

**DOI:** 10.1371/journal.pmed.1002324

**Published:** 2017-07-05

**Authors:** Peter Boone, Alex Eble, Diana Elbourne, Chris Frost, Chitra Jayanty, Rashmi Lakshminarayana, Vera Mann, Rohini Mukherjee, Gilda Piaggio, Padmanabh Reddy

**Affiliations:** 1 Effective Intervention, Centre for Economic Performance, London School of Economics, London, United Kingdom; 2 Teachers College, Columbia University, New York, New York, United States of America; 3 Medical Statistics Department, London School of Hygiene &Tropical Medicine, London, United Kingdom; 4 uMotif Digital Health, London, United Kingdom; 5 Freelance statistical consultant, London, United Kingdom; 6 Naandi Foundation, Hyderabad, India; 7 Statistika Consultoria, Divonne-les-Bains, France; 8 NICE Foundation, Hyderabad, India; Harvard University, UNITED STATES

## Abstract

**Background:**

In the mid-2000s, neonatal mortality accounted for almost 40% of deaths of children under 5 years worldwide, and constituted 65% of infant deaths in India. The neonatal mortality rate in Andhra Pradesh was 44 per 1,000 live births, and was higher in the rural areas and tribal regions, such as the Nagarkurnool division of Mahabubnagar district (which became Nagarkurnool district in Telangana in 2014). The aim of the CHAMPION trial was to investigate whether a package of interventions comprising community health promotion and provision of health services (including outreach and facility-based care) could lead to a reduction of the order of 25% in neonatal mortality.

**Methods and findings:**

The design was a trial in which villages (clusters) in Nagarkurnool with a population < 2,500 were randomised to the CHAMPION package of health interventions or to the control arm (in which children aged 6–9 years were provided with educational interventions—the STRIPES trial). A woman was eligible for the CHAMPION package if she was married and <50 years old, neither she nor her husband had had a family planning operation, and she resided in a trial village at the time of a baseline survey before randomisation or married into the village after randomisation. The CHAMPION intervention package comprised community health promotion (including health education via village health worker–led participatory discussion groups) and provision of health services (including outreach, with mobile teams providing antenatal check-ups, and facility-based care, with subsidised access to non-public health centres [NPHCs]). Villages were stratified by travel time to the nearest NPHC and tribal status, and randomised (1:1) within strata. The primary outcome was neonatal mortality. Secondary outcomes included maternal mortality, causes of death, health knowledge, health practices including health service usage, satisfaction with care, and costs. The baseline survey (enumeration) was carried out between August and November 2007. After randomisation on 18 February 2008, participants, data collectors, and data analysts were not masked to allocation. The intervention was initiated on 1 August 2008. After an inception period, the assessment start date was 1 December 2008. The intervention ended on 31 May 2011, and data collection was completed on 30 November 2011. Primary analyses followed the intention to treat principle. In all, 14,137 women were enrolled in 232 control villages, and 15,532 in 232 intervention villages. Of these, 4,885 control women had 5,474 eligible pregnancies and gave birth to 4,998 eligible children. The corresponding numbers in intervention villages were 5,664 women, 6,351 pregnancies, and 5,798 children. Of the live-born babies, 343 (6.9%) in the control arm and 303 (5.2%) in the intervention arm died in their first 28 days of life (risk ratio 0.76, 95% CI 0.64 to 0.90, *p =* 0.0018; risk difference −1.59%, 95% CI −2.63% to −0.54%), suggesting that there were 92 fewer deaths (95% CI 31 to 152) as a result of the intervention. There were 9 (0.16%) maternal deaths in the control arm compared to 13 (0.20%) in the intervention arm (risk ratio 1.24, 95% CI 0.53 to 2.90, *p =* 0.6176; 1 death was reported as a serious adverse event). There was evidence of improved health knowledge and health practices including health service usage in the intervention arm compared to the control arm. Women in the intervention arm were more likely to rate their delivery and postnatal care as good or very good. The total cost of the CHAMPION interventions was US$1,084,955 ($11,769 per life saved, 95% CI $7,115 to $34,653). The main limitations of the study included that it could not be masked post-randomisation and that fetal losses were not divided into stillbirths and miscarriages because gestational age was not reliably reported.

**Conclusions:**

The CHAMPION trial showed that a package of interventions addressing health knowledge and health seeking behaviour, buttressing existing health services, and contracting out important areas of maternal and child healthcare led to a reduction in neonatal mortality of almost the hypothesized 25% in small villages in an Indian state with high mortality rates. The intervention can be strongly justified in much of rural India, and is of potential use in other similar settings. Ongoing changes in maternal and child health programmes make it imperative that a similar intervention that establishes ties between the community and health facilities is tested in different settings.

**Trial registration:**

ISRCTN registry ISRCTN24104646

## Abstract 2

In CHAMPION, a cluster-randomized trial carried out in rural India, Diana Elbourne and colleagues investigate the benefit of a package of interventions for neonatal care.

## Author summary

**Why was this study done?:**

- At the time this study was planned, the rate of death of children in the first 28 days of life was very high in parts of rural India.
- Earlier research had suggested that community health promotion, along with buttressing existing maternity and neonatal services, might reduce this rate.

**What did the researchers do and find?:**

- We randomised small villages in the Nagarkurnool district in Telangana either to the package of health interventions or to control.
- We provided married women in the intervention villages with community health promotion (including health education via village health worker–led participatory discussion groups), outreach (including mobile teams providing antenatal check-ups), and facility-based care (including subsidised access to non-public health centres).
- We enrolled 14,137 women in 232 control villages and 15,532 in 232 intervention villages. In all, 4,885 control women had 5,474 eligible pregnancies and gave birth to 4,998 eligible children. The corresponding numbers in intervention villages were 5,664 women, 6,351 pregnancies, and 5,798 children.
- We found that, of the live-born babies, 343 (6.9%) in the control arm and 303 (5.2%) in the intervention arm died in their first 28 days of life, suggesting that there were 92 fewer deaths as a result of the intervention.
- We also found evidence of improved health knowledge and health practices including health service usage in the intervention arm compared to the control arm.

**What do these findings mean?:**

- The research findings mean that interventions such as those in the CHAMPION trial can be strongly justified in much of rural India.
- Further research is needed to assess whether the interventions are of use in other rural settings with similar socioeconomic and cultural patterns, similar neonatal mortality rates, and the existence of some private health provision.

## Introduction

Worldwide, nearly 40% of deaths in children under 5 years occur in the neonatal period (first 28 days of life) [1–3]: over 2.8 million neonates died in 2010 [4]. A review of global progress on the Millennium Development Goals found that for goal 4 (reducing child mortality), mortality in children under 5 years fell by 28% between 1990 and 2008, but reductions in neonatal mortality remained slow [5,6].

In India, 1.68 million children aged under 5 years died in 2010, with 52% of the deaths occurring in the first 28 days [1]—a neonatal mortality rate (NMR) of 39/1,000 live births [7]. Worldwide, India contributed nearly 25% of neonatal deaths [6], and NMRs in many regions of India remained near the highest rates worldwide [8]. Three-quarters of neonatal deaths in India were attributed to prematurity, low birthweight, neonatal infections, birth asphyxia, and birth trauma [9]. These deaths often occurred in rural environments, where children are commonly born at home and, while healthcare facilities were available, their quality could be inadequate [10]; in addition, poor health knowledge and practices including health service usage may be contributory factors [6].

Studies [8] have shown the potential for reducing neonatal deaths through providing home-based neonatal care, including management of sepsis [11]; behaviour change interventions with a focus on hypothermia [12]; community mobilisation through participatory discussion groups (PDGs) [13]; and implementation of the Indian Integrated Management of Neonatal and Childhood Illness programme for neonates born at home, which provides improved treatment of illness for children and home visits for newborn care [14]. An additional strategy in the developing world has been to use health providers not affiliated with the government to deliver services, particularly in areas where government service delivery may be underperforming [10]. Evidence about the effectiveness of such strategies is scarce [15–17].

The *Lancet* Neonatal Survival Series recommended future studies implement and evaluate packages of care that provide family, community, outreach, and facility-based care [18]. A subsequent systematic review [19] concluded that ‘the empirical evidence for the impact of neonatal health care packages is a weak base on which to build effective programmes…. An evidence-based approach to packaging interventions is clearly needed but requires substantial investment in high-quality research’.

The CHAMPION cluster randomised controlled trial (RCT) was initiated in 2007 to evaluate such a package of interventions, comprising community health promotion (including health education delivered via village health worker [VHW]–led PDGs) and provision of health services including outreach (mobile teams providing antenatal check-ups) and facility-based care (specifically subsidised access to pregnancy-related care provided by non-public health centres [NPHCs]). The primary hypothesis was that this package could reduce neonatal mortality by 25%. A cluster RCT design was needed as the interventions were delivered at the village level. An important element was an integral economic evaluation.

Around the time the trial was being designed and implemented, the Indian government introduced a number of maternal and child health programmes, including incentives to encourage institutional deliveries and care. One of the key elements of these programmes is the involvement of the Accredited Social Health Activists (ASHAs), who were trained to refer expectant women to medical institutions for delivery, and who also received incentives accordingly. These initiatives were not, however, fully established in most of the CHAMPION villages until well after the end of the trial (see Methods and Discussion).

The CHAMPION trial was implemented in Nagarkurnool district, Telangana. This was then Nagarkurnool division, Mahabubnagar district, in the state of Andhra Pradesh (AP). AP had one of the highest NMRs in India at the time, with 35% of neonatal deaths among children who were premature or of low birthweight [20]. An additional 14.1% of neonatal deaths involved neonatal infections, and 21.7% involved birth asphyxia and/or birth trauma [5,21,22]. The estimated NMR in rural regions of AP was between 49.2 [7] and 33 per 1,000 live births [23]. Mahabubnagar was among the 100 most disadvantaged districts in the country [24]; it was 90% rural [25] and had low levels of female literacy [26]. Nagarkurnool had a high tribal population [25,27] and a particularly high mortality rate [28]. Since June 2014, this division has been part of Telangana.

The aim of the CHAMPION trial was to investigate whether a package of interventions comprising community health promotion and provision of health services including outreach and facility-based care could lead to a reduction of the order of 25% in neonatal mortality.

## Methods

### Research ethics approvals

Approvals were obtained from the institutional review board of the L V Prasad Eye Institute, Hyderabad, India (number LEC07002; February 2007) and from the ethics committee of the London School of Hygiene & Tropical Medicine (LSHTM) (number 5166; June 2007).

The methods have been detailed [29], and are summarised below.

### Eligibility

#### Clusters

The CHAMPION trial was conducted in 464 villages (clusters) in Nagarkurnool with a population below 2,500, as larger villages generally have better access to health services, lower neonatal mortality rates, and therefore less potential to benefit from the interventions.

#### Women

A list of eligible women in each village was compiled from a baseline survey (enumeration) conducted before randomisation. A woman was eligible at enumeration if she was married and aged less than 50 years, neither she nor her husband had had a family planning operation, she was resident in the village at the time of the baseline survey, and she fulfilled the criteria for informed consent described below.

For analysis, women were not added, moved from one village to another, or removed from the list as a result of either temporary or permanent migration from the village where they were initially registered. The only permitted additions to enrolment were women who newly married into a trial village after enumeration (as ascertained in the monthly visits by the enumerators) and who were enrolled at that point.

#### Children

The protocol specified that children born to eligible women were to be included in the primary trial analysis if their estimated delivery at enumeration was at least 6 months after randomisation, to allow time for the interventions to be implemented and to have an impact on neonatal mortality. In fact, the intervention took longer than anticipated to implement, and a decision was taken by the trial steering committee (TSC) in autumn 2008 (without access to outcome data) to delay the date from which data would contribute to the analysis to 1 December 2008 (9.5 months after randomisation, and 4 months after first initiation of the intervention).

### Consent

State approval was obtained from the Andhra Pradesh Department of Health & Family Welfare. Village consent was obtained from each panchayat (smallest unit of democratically elected government in rural India). The trial protocol was explained to villagers in their local language. Oral consent was given by the panchayat and sarpanch (panchayat leader) during a meeting before randomisation, with written documentation of approval by the sarpanch. Women provided oral consent at enumeration. Women who married into a trial village post-randomisation were informed about the trial, and their consent obtained.

### Randomisation

The villages were stratified by travel time to the nearest designated NPHC (less than or greater than 1 hour) and tribal status (whether the village was a thanda [tribal village 2–3 km from the main village, with around 15 families], a penta [tribal village 20–30 km from the main village, with around 4–5 families], or non-tribal). Within each stratum, half the villages were allocated to the intervention arm and half to the control arm by the LSHTM statistician using computer-generated random numbers.

### Masking

After randomisation, participants, data collectors, and data analysts were not masked to allocation. Data collectors could not be masked as they were going into villages where the interventions were obvious and collecting data from participants who were not masked. Given this necessary lack of masking in the field, we felt that trying to ensure the masking of data analysts would add complexity for little benefit. The regular communication between the data analysts and the data collectors would have made such masking very difficult to implement. Further, the anticipated large difference in health knowledge between the arms would have made the randomisation clear.

### Interventions

#### Intervention villages

A package consisting of community health promotion and provision of health services was delivered in the intervention villages. The intervention teams were selected and managed by the NICE (Neonatal Intensive Care and Emergencies) Foundation (http://nicefoundation.in/about.html). Members of the intervention team were responsible for all service delivery activities in the intervention villages. Team members included a programme coordinator who led the team and coordinated care with the NPHCs; 2 programme officers who managed the field supervisors, midwives, and VHWs; field supervisors who were responsible for community mobilisation, mainly via PDGs, and for monitoring the VHWs; and midwives and VHWs. Regular intervention team meetings were held to share lessons learned and for future planning.

#### Health promotion activities

Each intervention village received a health education campaign during the trial. Folk culture (Kalajatha) in the form of song and dance was used to promote key maternal and child health themes. The intervention team worked with a group of artists for a month to finalise the messages and ways to disseminate them. They produced the final format after 2 pilot sessions in villages. The songs carried messages about safe maternal and child health practices, focusing on clearing misconceptions about maternal and child health, danger signs when pregnancies go wrong, trained birth assistance, safe motherhood, newborn care, the advantages of institutional delivery, the importance of fixed day health services (FDHSs), and the PDGs.

PDGs were conducted at least monthly and, in contrast to the health education campaign, primarily targeted women to improve mothers’ and caretakers’ health knowledge, increase each community’s awareness of maternal and child health, and encourage greater use of available health services, safe delivery techniques, and referral of high-risk women and babies to more specialist centres. The PDGs also gave mothers a forum to discuss solutions to important maternal and newborn health issues. Ten key discussion themes promoting good health practices including health service usage during and after pregnancy were repeated over the duration of the trial. These included recognising risk factors for baby and mother, drawing up birth plans, safe delivery techniques, family health, and immunisation. Other issues that were important in the villages were identified and delivered as a session. There was 1 PDG session (module) per theme. Posters, flip charts, and flash cards were used, supplemented by examples of actual foodstuffs at the nutrition session. Eligible women and other members of the community could attend the sessions, which happened on the day of the FDHS. Each session lasted approximately an hour and took place at a village venue suitable to most women. Sessions contained icebreakers, a review of the previous meeting, and new themes, with reinforcement through activities, demonstrations, and games.

Approximately 230 women were selected in consultation with the community and trained to serve as VHWs. Although an option in the protocol was for VHWs to be auxiliary nurse midwives (ANMs) or traditional birth attendants, in practice none were. They were resident women from or marrying into the village (‘daughter-in-laws of the village’), capable of reading and writing in Telugu. Most had a 10th grade education.

#### Health service activities

The aim of the CHAMPION trial interventions was not to run a parallel health service system but to strengthen existing government services, and so, where possible, the trial intervention team converged with the government flagship programmes, for instance, utilising ANM services to ensure tetanus vaccinations for pregnant women and administering child vaccinations through the government ANMs. However, many of the government initiatives were not in place in the trial villages during the period of the trial (see Discussion). In their absence, VHWs were the primary contact point for the intervention team in the village, responsible for delivering antenatal and health education in their allocated villages. Their major role was to identify and track eligible women in their village, visiting the houses of pregnant women and newborns, and documenting the events that occurred. They counselled pregnant women and their family members, mobilised pregnant women to attend the FDHS, supported midwives during the FDHS and provided follow-up care as advised by nurse midwives, provided case briefings to the midwife at each FDHS consultation, and supported the FDHS team in mobilising target audiences to PDG sessions. The VHWs were also involved in preparation for, and conducting or assisting at, normal deliveries in the village and providing follow-up care for postnatal women, including 3 visits during the first week, and 2 visits per week from the second to fourth week. An important role involved tracking high-risk mothers (identified based on vaginal bleeding; headache; persistent fever; abdominal cramps; dizziness; loss of consciousness or blurring of vision; convulsions; shakes; shivering; poor circulation and puffiness of the face, hands, or feet; lack of fetal movements over 4 hours; sudden weight gain or loss; previous history of fetal loss; obstructed labour; or signs of malaria; S1 Text), as well as detecting danger signs in newborns and facilitating timely referral and transport of pregnant woman or newborns, if needed. VHWs were provided with mucous suckers, mackintosh sheets, gauze pieces, rolls of cotton, brushes and antiseptic lotions (povidone-iodine for cord care), birthing kits, weighing scales, measuring tapes, and fetoscopes. VHWs received payment proportional to village size, to a maximum of 1,100 rupees for villages with close to 2,500 inhabitants.

VHWs underwent a 1-week residential training followed by quarterly 1-day refresher training. The training topics included anaemia and complaints in pregnancy such as fever, persistent headache, abdominal pain, and vaginal bleeding. VHWs were trained to monitor potential breast problems, oedema, fetal heartbeat, and fetal movements. They were taught to clinically assess fetal position, abnormal presentations, fundal height, and abnormal observations. The curriculum topics also included postnatal care, conducting safe home deliveries, home-based newborn care practices, recognising danger signs in pregnant women and newborns, and arranging referrals. VHWs were taught how to monitor and document implementation of the intervention, and how to conduct PDG sessions. Training kits included manuals, forms, flip charts, and delivery kits. Regular refresher sessions were held during the monthly meetings, which reviewed any issues raised in the previous PDG meetings.

Sixteen qualified midwives visited intervention villages regularly throughout the trial. Three were based at an NPHC. All midwives underwent a 10-day residential theoretical and practical training conducted by experts in maternal and neonatal health. Every fortnight, teams of 2 midwives (and a field supervisor) visited intervention villages and delivered the FDHS. One midwife delivered the antenatal and postnatal health checks, including pregnancy confirmation; assessment of fetal position; fundal height; gestational age; vaginal examination; abnormal observations from physical examination and investigations such as haemoglobin and urine analysis (sugar and albumin); and postnatal care and newborn care (head to foot examination). The other midwife monitored the delivery of health education by the VHW. Midwives also accompanied women with high-risk pregnancies to the NPHCs, conducted emergency deliveries at the village with the help of the VHWs, and, if needed in cases of complications, were responsible for referring the mother/neonate in distress to the next level of care accompanied by the VHW or the birth attendant.

Four NPHCs were identified to provide services that an expectant mother might require during pregnancy and for the first month of her baby’s life. The NPHCs had approximately 20 beds, with facilities and a qualified doctor for normal deliveries and cesarean sections and a paediatrician to see ‘problem’ newborns. NPHCs were selected based on criteria such as professional competence, existing infrastructure, and public perception of the facility. An initial contract of 3 months was agreed, after which favourable review led to a 6-month renewal. These NPHCs were expected to provide around-the-clock service, admission to the general ward, food for the patient and 1 attendant, and drugs and disposables to women from intervention villages. This contract was subject to a per-patient cost ceiling. A rigorous monitoring system implemented by the intervention team was part of the continuous quality assessment process that buttressed against overbilling and over-provision of NPHC services. Transport was not part of this contract, but 8 CHAMPION trial ambulances were provided by the NICE intervention team directly at the village level to pick up and drop off patients at the NPHCs. Women and their family members were also encouraged to use the emergency transport system provided by the state government [30]. However, this transport system was not operational at the trial start, and only inconsistently thereafter.

All eligible women in the intervention villages were issued a health card (S1 and S2 Figs) that tracked the various services they received under the programme, including immunisations, participation in health groups, regular check-ups, and hospitalisations.

#### Control villages

CHAMPION control villages were not offered the CHAMPION interventions. However, children between the ages of 6 and 9 years in 107 of the 232 CHAMPION control villages were provided with the Naandi Foundation’s Ensuring Children Learn (ECL) programme as part of a parallel trial (STRIPES [31]) utilising the same randomisation as in CHAMPION, but with CHAMPION controls receiving ECL. A village was eligible for inclusion in STRIPES if it was already participating in the CHAMPION trial, the village had at least 1 public primary school for both boys and girls, the school operated in the 2007–2008 academic year and was likely to continue operations during the following 2 years, and at least 15 children in total were present in classes 2, 3, and 4 in the school at the time of the baseline survey. Children were eligible for inclusion in the analysis of the trial if they were resident in an eligible village, they were recorded in the enumeration conducted in January 2008 as planning to be enrolled in class 2, 3, or 4 at the government school located in their village in the 2008–2009 academic year, and if, after hearing an explanation of the trial, their parent(s) or guardian(s) did not choose to opt out of the trial.

The remaining 125 CHAMPION control villages did not meet the eligibility requirements for STRIPES but were nevertheless offered ECL outside the trial. A key purpose of implementing ECL in the CHAMPION control villages was to give Naandi a presence in the control villages, hence helping to ensure that CHAMPION data collection was carried out to the same standard in intervention and control villages. We anticipated that the primary-school-level ECL educational intervention would not affect neonatal mortality in the control arm.

### Outcomes

#### Primary outcome

The primary outcome for the trial was neonatal mortality defined as death in the first 28 days of life of a live-born baby from a pregnancy (regardless of whether singleton or multiple) with expected delivery date (EDD) on or after the assessment start date.

#### Secondary outcomes

Secondary outcomes included age at neonatal death, and cause as classified by verbal autopsy (VA) [32,33], and maternal mortality, defined as the death of a woman during pregnancy or within 42 days of the end of pregnancy from any cause related to or aggravated by the pregnancy or its management, among pregnancies with EDD on or after the assessment start date (the cause was classified by VA [32]). Other outcomes included health knowledge (including risk symptoms for neonates), health service usage (pregnancy care, care at delivery, place of delivery, care after delivery), and satisfaction with care. The costs of the interventions per life year saved (LYS) and per neonatal death averted are discussed in the economic evaluation below. Neonatal and maternal morbidity were listed in the protocol as potential outcomes, but, for logistical reasons, these data were not collected. Death in, or in transit to, an NPHC was considered a serious adverse event.

### Sample size

Making the simplifying assumption that each village had an average population of 659 [28] with a birth rate of 23 per 1,000 population per year [25], 38 births per village over the trial period were expected. Assuming an intra-cluster correlation coefficient (ICC) of 0.00644 [34] for the primary outcome, 330 villages would give 80% power (5% 2-sided significance) to detect a 25% reduction in neonatal mortality from 4.38% [28] to 3.29%. To account for possible migration (e.g., to their mother’s village around 1 to 3 months before the EDD and/or during the summer to work in bigger towns) and for possible losses to follow-up, the target size was increased to 464 villages.

### Trial management

The trial was managed by a TSC and a trial management group (TMG). The TSC met periodically to discuss overall progress. The TMG met monthly to ensure efficient day-to-day running of the trial. The Naandi Foundation was the local trial coordinating centre and was responsible for the research components and data management in the field. The trial database was designed and supported by Effective Intervention. LSHTM was responsible for all statistical aspects. The NICE Foundation was subcontracted to implement the interventions.

### Data collection

To reduce bias, the research/data collection team was selected and managed by the Naandi Foundation and was independent of the intervention teams. While the research team could not be masked to the intervention status of a village, they were trained to collect data in an identical manner in the intervention and control villages. No data from the research team were used by the intervention team.

The research team monitored and collected data about pregnant women and their babies in both intervention and control villages. The team included a research coordinator who led the team and was responsible for timely data entry; a research officer who validated the collected data and monitored field staff; 19 data supervisors who monitored enumerators, supervised the data collection process, obtained detailed follow-ups on pregnancy outcomes, and collected information for VAs when necessary; 432 enumerators, to cover the 464 villages, who identified eligible women and tracked pregnancies; and data entry operators who were responsible for data entry and generation of monitoring forms.

In each village, the enumerator conducted a monthly pregnancy check. The data were then transported to Hyderabad, where all data were double-entered into a database, from which forms were printed for the next monthly monitoring and taken to the field. If a woman was identified as pregnant, the database triggered a follow-up form after the EDD. Data supervisors conducted a follow-up interview 6 weeks after delivery. Information about women who could not be located or were known to have migrated beyond the trial area was sought through their relatives in the village. If a live-born baby was suspected to have died aged 28 days or less, or a woman known to be pregnant was suspected to have died during pregnancy or within 42 days of the end of pregnancy, a specially trained data supervisor interviewed the family and completed a VA form to enable independent assessors to assign the primary cause of death. Data about health knowledge, health practices including health service usage, and satisfaction with care were collected by the research team for women with an eligible pregnancy.

Data supervisors visited enumerators monthly and cross-checked a sample of their reports. The supervisors and enumerators met monthly at regional headquarters to deposit monthly survey results, check that forms were properly completed, and discuss any problems in the field. Flags were created in the database to check on inconsistencies or missing data in a timely manner.

### Statistical analysis

Primary analysis of outcomes followed the intention to treat principle.

As this trial has a complex hierarchical structure, with multiple women per cluster, potentially multiple pregnancies per woman, and potentially multiple births per pregnancy, we used a generalised estimating equations (GEE) analysis approach [35]. This approach assumes non-independence of all observations from the same cluster, and so also accounts for non-independence of multiple outcomes from the same woman.

For the primary outcome, the relative risk [36] with a 95% confidence interval was obtained from a GEE model with a binary outcome, a log link, and a ‘working’ assumption of independence as recommended by Lee and Nelder [37], with robust standard errors to take account of clustering.

Although the protocol stated that we would not adjust for covariates in the primary analysis, in the final statistical analysis plan we amended this so that the model included the stratifying variables (travel time to nearest NPHC and tribal status of village). Secondary exploratory analyses extended the relative risk model by adding an interaction between treatment arm and timing of enrolment (pre- versus post-randomisation) to assess the possibility of bias in the primary outcome arising due to differential post-randomisation enrolment in the 2 arms. At the request of a reviewer, an additional stratified analysis by whether or not control villages were in the STRIPES trial was added.

The risk difference (and hence the estimated number of lives saved and the number needed to treat) was estimated using the same model (without an interaction), but with an identity rather than a log link. Although given as an option in the protocol, survival analysis was not used because the period when neonatal death was possible was very short and the precise date of death was not always known.

For secondary binary outcomes, relative risks were estimated using the same approach as for the primary outcome. Other outcomes are reported descriptively but without statistical testing due to the large number of comparisons.

Interim analyses were pre-specified and provided confidentially by the trial statisticians to the independent data monitoring committee (DMC), which was guided by the Haybittle–Peto rule [38].

### Economic evaluation

A cost-effectiveness calculation in terms of cost per neonatal death averted and cost per LYS was conducted. (Cost per disability-adjusted life year saved could not be considered as no measure of future disability was available.) The sensitivity of these outcomes to the most important inputs—labour costs, fuel costs, and exchange rate movements—was examined.

The direct additional provider costs of the CHAMPION intervention activities compared to existing standard of care in the control arm was measured. Total spending was cross-checked with funding sources for accuracy. Spending was divided into running costs and capital costs. There were very limited start-up costs, which were assumed to be fully depreciated during the trial, because the NICE Foundation had previously implemented a pilot version of the programme elsewhere in India. Straight line depreciation of capital equipment (computers, ambulances, refurbishment costs for public health centres, and medical equipment for these clinics based on 3-, 4-, 6-, and 8-year lifespans, respectively) was allowed for, consistent with usual account practices. Capital spending outside these items was depreciated immediately. There were no contributions in kind. Office facility costs, which were shared with other projects managed by the Naandi Foundation, were allocated to the project according to estimated share of usage (30%).

Annual cost figures were adjusted by India’s GDP deflator in order to convert values to May 2011 rupees. Average exchange rates from May 2011 were used to convert rupee figures to US dollars.

## Results

Enumeration was carried out between August and November 2007, with randomisation on 18 February 2008. After an inception period, the intervention was initiated from 1 August 2008. The assessment start date (see Methods) was 1 December 2008. The DMC met by teleconference to review interim analyses on 2 occasions but did not recommend early stopping. The intervention ended on 31 May 2011. Data collection was completed on 30 November 2011.

**Fig 1:**
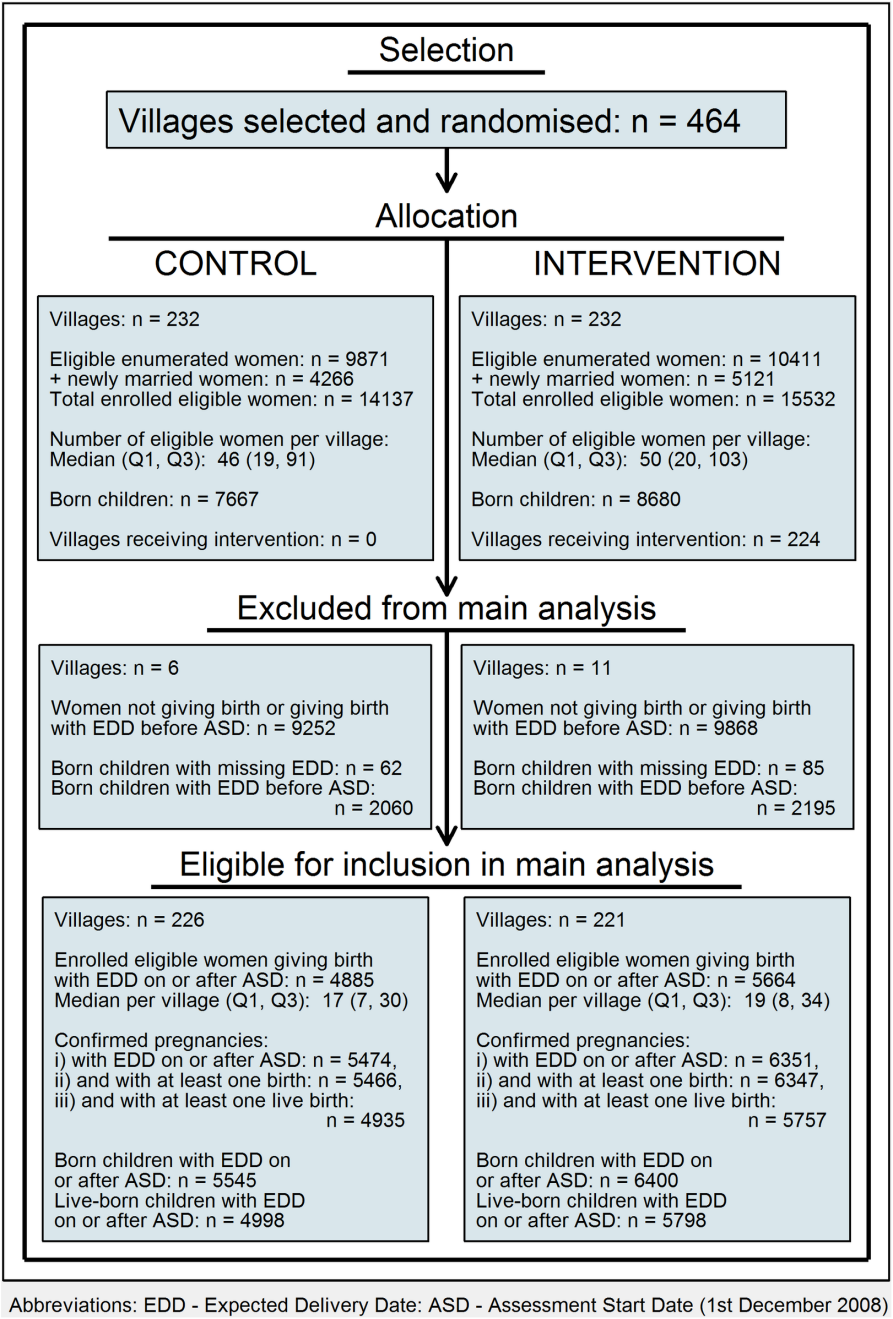
Flowchart: Clusters, women, and children at trial entry and in final analysis.

In the 464 villages, 20,282 enumerated women met the eligibility criteria (9,871 in control and 10,411 in intervention villages, a difference attributable to chance). Non-eligibility was mainly due to sterilisation. A further 9,387 newly married women were enrolled in the trial villages—more in intervention villages (4,266 control, 5,121 intervention). The total number of enrolled eligible women was thus 14,137 control and 15,532 intervention (Fig 1; S1–S4 Datas).

Based on all 464 villages, the number of births per village varied between 0 and 141 (median = 28, interquartile range [IQR] Q1 = 12, Q3 = 49); 17 villages (6 control, 11 intervention) had no live births during the assessment period (3 had stillbirths but no live births, and the other 14 had either only miscarriages or abortions or had no confirmed pregnancies [where a confirmed pregnancy was defined as either a live birth or a death where the pregnancy was confirmed by verbal autopsy]). Based on the 447 villages with at least 1 live birth, the number of live births per village varied between 1 and 92 (median = 21, IQR Q1 = 9, Q3 = 37) in the intervention villages and between 1 and 98 (median = 18, IQR Q1 = 7, Q3 = 31) in the control villages. In all, 4,885 (34.6%) of the control village women had 5,474 confirmed pregnancies with an EDD of 1 December 2008 or later, and gave birth to 4,998 eligible children. The corresponding numbers in the intervention villages were 5,664 (36.5%) women, 6,351 pregnancies, and 5,798 children.

Comparability of control and intervention villages is shown in Table 1 (see also S1 and S5–S7 Datas). None of the control villages and 224 of the 232 intervention villages received at least some of the intervention. There were no or very few women residing permanently in the 8 remaining villages due to seasonal migration specific to the tribal communities.

**Table 1:** Comparability between trial arms.

Characteristic	Subcategory	Arm	
		Control	Intervention
**Villages**		*N =* 232	*N =* 232
Tribal status, *n* (percent)	Non-tribal	136 (58.6)	135 (58.2)
	Penta	21 (9.1)	20 (8.6)
	Thanda	75 (32.3)	77 (33.2)
Travel time to NPHC, *n* (percent)	<60 minutes	176 (75.9)	176 (75.9)
	>60 minutes	56 (24.1)	56 (24.1)
**Women giving birth during the period of the trial (with EDD on or after the ASD)**		*N =* 4,885	*N =* 5,664
Age of woman (years), mean (SD)		21.2 (3.9)	21.3 (3.9)
Education of woman, *n* (percent)	Missing	304	322
	No education	2,654 (57.9)	3,146 (58.9)
	1–4 years	465 (10.2)	457 (8.6)
	5–7 years	527 (11.5)	646 (12.1)
	8+ years	935 (20.4)	1,093 (20.5)
Education of spouse, *n* (percent)	Missing	403	431
	No education	2,009 (44.8)	2,336 (44.6)
	1–4 years	326 (7.3)	378 (7.2)
	5–7 years	695 (15.5)	823 (15.7)
	8+ years	1,452 (32.4)	1,696 (32.4)
Previous miscarriage, *n* (percent)		1,157 (25.3)	1,372 (25.7)
Neonatal deaths in the last year, *n* (percent based on births in the last year)		59 (5.8) (*N* = 1,023)	74 (6.7) (*N* = 1,106)
**Pregnancies during the period of the trial resulting in a birth (with EDD on or after the ASD)**		*N =* 5,466	*N =* 6,347
Multiplicity of birth, *n* (percent)	Singleton	5,388 (98.6)	6,295 (99.2)
	Twin	77 (1.4)	51 (0.8)
	Triplet	1 (0.02)	1 (0.02)
**Children born during the period of the trial (with EDD on or after the ASD)**		*N =* 5,545	*N =* 6,400
Sex, *n* (percent)	Male	2,552 (46.0)	3,011 (47.0)
	Female	2,446 (44.1)	2,788 (43.6)
	Not known	547 (9.9)	601 (9.4)

ASD, assessment start date; EDD, expected delivery date; NPHC, non-public health centre.

Between 1 August 2008 and 31 May 2011, 9,939 PDGs were held in the intervention villages, at least monthly, and 12,195 FDHSs were conducted during the trial. The total number of pregnant women who attended the FDHSs was 4,492, against a potential number of 5,022.

Of the 4 NPHCs, 2 were managed by local private doctors, and 1 by midwives hired by the intervention team (with paediatric support available); the contract with the fourth was terminated due to quality concerns after supervisory visits, and the team referred cases to another NPHC. Over this period, there were 913 referrals (526 maternal and 387 neonatal—290 from a village to an NPHC or government facility, 429 from an NPHC to a tertiary care facility, and 194 from one NPHC to another or from a government facility to an NPHC or to a tertiary care centre); 24% of deliveries in the intervention arm occurred at one of the NPHCs, 21% at home, 29% at government-related facilities, and 28% at private facilities. In the control villages, 21% of deliveries were at home, 30% at government-related facilities, and 47% at private facilities.

Of the 11,945 children with an EDD of 1 December 2008 or later, 1,149 (602 in the intervention arm, 547 in the control arm) were stillborn or died earlier in the pregnancy. Of the live-born babies, 343 out of 4,998 (6.9%) in the control arm died in their first 28 days of life, and 303 out of 5,798 (5.2%) in the intervention arm (risk ratio 0.76, 95% CI 0.64 to 0.90, *p* = 0.0018; Table 2; S1, S9 and S10 Datas; risk difference −1.59%, 95% CI −2.63% to −0.54%), suggesting 92 fewer deaths (95% CI 31 to 152) as a result of the intervention. The ‘number needed to treat’ to prevent 1 neonatal death was 63 (95% CI 38 to 185). The babies in the control arm were more likely to die in the first week of life, but there were no clear differences between trial arms in terms of sex or cause of death.

**Table 2:** Neonatal mortality for births with expected delivery date after the assessment start date.

Outcome	Control arm (*N =* 5,545)	Intervention arm (*N =* 6,400)	Risk ratio (95% CI)
**Dead at birth or pregnancy loss**	547 (9.9)	602 (9.4)	
**Alive at birth**	4,998 (90.1)	5,798 (90.6)	
Alive at 28 days	4,655 (93.1)	5,495 (94.8)	
Neonatal death	343 (6.9)	303 (5.2)	0.76 (0.64 to 0.90)
**Neonatal death: age at death**			
Within first day	83 (24.2)	65 (21.5)	
1–7 days	189 (55.1)	156 (51.5)	
8–28 days	56 (16.3)	63 (20.8)	
Not known	15 (4.4)	19 (6.3)	
**Neonatal death: gender**			
Female	156 (45.5)	147 (48.5)	
Not known	5 (1.5)	4 (1.3)	
**Neonatal death: cause of death**			
Congenital anomaly	31 (9.0)	21 (6.9)	
Multiple birth	26 (7.6)	13 (4.3)	
Maternal disease	44 (12.8)	32 (10.6)	
Specific fetal condition	4 (1.2)	3 (1.0)	
Small-for-date infant	19 (5.5)	15 (5.0)	
Placental abruption	3 (0.9)	2 (0.7)	
Obstetric complication	59 (17.2)	54 (17.8)	
Specific infant condition	61 (17.8)	63 (20.8)	
Asphyxia	40 (11.7)	31 (10.2)	
Immaturity	33 (9.6)	31 (10.2)	
Not known	23 (6.7)	38 (12.5)	

Data are given as *n* (percent).

In exploratory analyses the effect of the intervention on neonatal mortality did not differ significantly by travel time to the nearest NPHC (interaction test *p* = 0.6516) or by tribal status of village (interaction test *p* = 0.3538). Nor was there a significant interaction by whether the village participated in the STRIPES education trial (interaction *p* = 0.2876) or according to timing of enrolment (investigated to assess the possibility of bias in the primary outcome due to differential post-randomisation enrolment between the 2 arms; pre-randomisation versus newly married post-randomisation, *p* = 0.1552). Table 3 shows the results in these strata (see also S1 and S11 Datas).

**Table 3:** Neonatal mortality according to characteristics of villages/women for births with expected delivery date after the assessment start date.

Characteristics	Control arm	Intervention arm	Risk ratio (95% CI)
**Travel time from village to nearest NPHC**			
<60 minutes	289/4,200 (6.9)	252/4,868 (5.2)	0.75 (0.62, 0.90)
>60 minutes	54/798 (6.8)	51/930 (5.5)	0.84 (0.53, 1.33)
**Tribal status of village**			
Non-tribal	260/3,995 (6.5)	237/4,652 (5.1)	0.78 (0.64, 0.95)
Penta	12/93 (12.9)	6/116 (5.2)	0.39 (0.17, 0.91)
Thanda	71/910 (7.8)	60/1,030 (5.8)	0.74 (0.51, 1.08)
**STRIPES trial participation**			
Village in STRIPES trial	226/3,502 (6.5)	216/4,145 (5.2)	0.81 (0.67, 0.99)
Village not in STRIPES trial	117/1,496 (7.8)	87/1,653 (5.3)	0.66 (0.48, 0.91)
**Timing of enrolment**			
Woman recruited pre-randomisation	221/3,368 (6.6)	200/3,697 (5.4)	0.83 (0.67, 1.02)
Woman recruited post-randomisation	122/1,630 (7.5)	103/2,101 (4.9)	0.65 (0.49, 0.86)

Data are given as *n/N* (percent).

NPHC, non-public health centre.

The estimated design effect for neonatal mortality was 1.306. There are complexities with defining an ICC for neonatal mortality in this study. The ICC has a simple definition only for a continuous outcome with a 2-level hierarchy. Here there is a 4-level hierarchy (potentially multiple children per pregnancy, multiple pregnancies per woman, and multiple women per village), and the outcome is binary (even for a 2-level hierarchy there are a number of different definitions of the ICC for a binary outcome [39], which can differ quite markedly). Ignoring these difficulties and estimating the ICC from the design effect and the mean number of live births per village (25.74) via the usual formula gives an estimated ICC of 0.012.

Table 4 shows maternal mortality based on pregnancies with an EDD of 1 December 2008 or later (see also S1 and S12 Datas). There were 9 (0.16%) deaths in the control arm compared to 13 (0.20%) in the intervention arm (risk ratio 1.24, 95% CI 0.53 to 2.90, *p* = 0.6176). In both arms, the main causes were haemorrhage and hypertensive disorders, although there were also cases of sepsis, obstructed labour, and other pregnancy-related conditions in the intervention arm.

**Table 4:** Maternal mortality for pregnancies with expected delivery date after the assessment start date.

Outcome	Arm		Risk ratio (95% CI)
	Control (*N =* 5,474)	Intervention (*N =* 6,351)	
**Maternal deaths, *n* (percent)**	9 (0.1644)	13 (0.2047)	1.24 (0.53 to 2.90)
**Cause of death, *n***			
Haemorrhage	5	3	
Sepsis	0	2	
Hypertensive disorders	4	3	
Obstructed labour	0	3	
Other pregnancy-related conditions	0	2	

### Harms potentially related to the CHAMPION intervention

One serious adverse event was reported in October 2008. Following a trial of labour at an NPHC, a woman died after transfer in a CHAMPION trial ambulance to a private hospital on her family’s wishes.

### Health knowledge, health practices including health service usage, and satisfaction with care

Based on women with pregnancies that resulted in a live birth with EDD on or after the assessment start date, a higher proportion of women in the intervention arm than in the control arm gave correct answers on 20 out of 23 health knowledge items when asked about risk symptoms for neonatal problems (Table 5; S1 and S13 Datas).

**Table 5:** Mothers’ knowledge of risk symptoms for neonates (based on pregnancies that resulted in a live birth with estimated delivery date on or after the assessment start date).

**Health knowledge item**	**Control arm (*N =* 4,935)**	**Intervention arm (*N =* 5,757)**	
**List signs of anaemia**^**1**^			
Pale nails	1,116 (22.6)	1,903 (33.1)	
Pale palms	779 (15.8)	1,281 (22.3)	
Pale eyelids	1,188 (24.1)	1,936 (33.6)	
Tiredness	1,469 (29.8)	1,600 (27.8)	
Missing	3 (0.06)	3 (0.05)	
**List signs of an emergency delivery**^**1**^			
Breech	359 (7.3)	988 (17.2)	
Water broke	529 (10.7)	885 (15.4)	
Heavy bleeding	277 (5.6)	438 (7.6)	
Green or brown waters	56 (1.1)	66 (1.2)	
Mother has fever	94 (1.9)	213 (3.7)	
Missing	3 (0.08)	3 (0.05)	
**Options if baby develops diarrhoea**		
Breastfeed more frequently and try to make the baby drink more^2^	1,277 (25.9)	1,498 (26.1)
Breastfeed when the child would like to, but don’t make any special efforts	2,673 (54.2)	3,099 (53.9)
Wait for a short while until the diarrhoea settles down and then breastfeed again	481 (9.8)	516 (9.0)
Wait for quite a long time until you are sure the diarrhoea has stopped and then breastfeed again	280 (5.7)	406 (7.1)
Missing	7 (0.14)	10 (0.17)
**List signs of dehydration in newborn**^**1**^		
Reduced urinating	50 (1.0)	138 (2.4)
Reduced tears	76 (1.5)	137 (2.4)
Dry mouth and lips	654 (13.3)	902 (15.7)
Sunken eyes	1,589 (32.2)	1,793 (31.2)
Sunken fontanelle	55 (1.1)	111 (1.9)
Missing	3 (0.06)	3 (0.05)
**List signs of neonatal sepsis**^**1**^		
Reduced sucking	13 (0.3)	34 (0.6)
Drowsy or unconscious	8 (0.2)	22 (0.4)
Cold to touch	8 (0.2)	14 (0.2)
Fever	358 (7.3)	666 (11.6)
Breathing fast	252 (5.1)	441 (7.7)
Chest is indrawing	10 (0.2)	35 (0.6)
Grunting	138 (2.8)	404 (7.0)
Skin infection or infection in umbilical cord	194 (3.9)	281 (4.9)
Missing	3 (0.06)	3 (0.05)

Data given as the number (percent) of respondents stating a given sign or course of action.

^1^Not mutually exclusive options; all correct responses are listed. These questions were free response, and women answered only what they knew without prompting.

^**2**^This is the correct option.

Women with pregnancies with EDD on or after the assessment start date were asked about antenatal, delivery, and postnatal care experiences (although it was usually felt insensitive to ask these question of women whose deliveries did not lead to a live birth). Women in the intervention arm were more likely to have attended more antenatal care, with more testing and advice; have trained personnel at delivery; deliver in an institution; have antiseptic dressing for the umbilical cord; have their baby wiped and weighed; initiate breastfeeding soon after birth; and have trained personnel for postnatal checks (Table 6; S1 and S14 Datas). Women in the intervention arm were more likely to rate their delivery and postnatal care as good or very good (Table 7; S1 and S15 Datas).

**Table 6:** Antenatal, delivery, and postnatal care experience of women with pregnancies with estimated delivery date on or after the assessment start date.

Question	Answer	Control arm (*N =* 5,474)	Intervention arm (*N =* 6,351)
**How many antenatal visits did you have?**	1 to 5	1,619 (33.2)	593 (12.1)
	6 to 10	2,275 (46.6)	1,890 (38.7)
	≥11	988 (20.3)	3,252 (66.6)
	Missing	63	29
	Not asked^1^	529	587
**Were you weighed?**	Yes	4,354 (89.2)	5,628 (98.2)
	Missing	64	30
	Not asked^1^	530	587
**Was your blood pressure taken?**	Yes	4,613 (94.5)	5,687 (99.2)
	Missing	64	30
	Not asked^1^	530	587
**Did you give a urine sample?**	Yes	4,424 (90.6)	5,572 (97.2)
	Missing	64	30
	Not asked^1^	529	587
**Did you give a blood sample?**	Yes	4,422 (90.6)	5,562 (97.0)
	Missing	64	30
	Not asked^1^	530	587
**Was your abdomen checked?**	Yes	4,608 (94.4)	5,676 (99.0)
	Missing	64	30
	Not asked^1^	530	587
**Were you told your delivery date?**	Yes	4,193 (85.9)	5,295 (92.3)
	Missing	64	30
	Not asked^1^	530	587
**Did you receive a tetanus shot?**	Yes	4,811 (97.5)	5,664 (98.4)
	Missing	10	6
	Not asked^1^	528	587
**Did you receive iron/folic acid?**	Yes	4,758 (96.4)	5,649 (98.1)
	Missing	10	5
	Not asked^1^	528	587
**Who assisted delivery?**^**2**^	Doctor	2,278 (46.2)	2,796 (56.7)
	ANM/nurse/midwife/LHV	3,618 (73.4)	4,453 (90.3)
	Other health personnel	159 (3.2)	247 (5.0)
	Traditional birth attendant	918 (18.6)	971 (19.7)
	Friend/relative	2,351 (47.7)	2,485 (50.4)
	Other	400 (8.1)	365 (7.4)
	Nobody	9 (0.2)	7 (0.1)
	Missing	11	6
	Not asked^1^	533	592
**Where did you give birth?**	Home (yours, your parents, other)	1,053 (21.4)	1,022 (17.8)
	Public facility	1,512 (30.5)	1,689 (29.3)
	Private facility	2,315 (47.0)	1,615 (28.1)
	CHAMPION NPHC	2 (0.04)	1,362 (23.6)
	Other	47 (1.0)	65 (1.1)
	Missing	11	6
	Not asked^1^	534	592
**Did you arrive before, during, or after delivery?**	Before	552 (14.4)	615 (13.2)
	During	3,276 (85.6)	4,047 (86.8)
	After	1 (0.0)	2 (0.0)
	Missing	1,109	1,094
	Not asked^1^	536	593
**What transport did you have to the facility?^2^**	CHAMPION trial ambulance	5 (0.1)	466 (12.2)
	Paid private transportation	2,795 (73.0)	2,948 (77.0)
	Emergency government transport	1,044 (27.3)	1,422 (37.1)
	Walked	134 (3.5)	217 (5.7)
	Other	39 (1.0)	39 (1.0)
	Missing	1,109	1,092
	Not asked^1^	535	593
**What was the mode of delivery?**	Vaginal cephalic	3,571 (72.3)	4,087 (71.0)
	Vaginal breech	6 (0.1)	10 (0.2)
	Cesarean section	1,357 (27.5)	1,656 (28.8)
	Instrumental (forceps/vacuum)	2 (0.0)	2 (0.0)
	Missing	9	7
	Not asked^1^	529	589
**What was used to cut the umbilical cord?**	Knife	5 (0.3)	3 (0.2)
	Scissors	345 (19.9)	373 (20.0)
	Razor (new)	1,348 (78.0)	1,452 (78.0)
	Sickle	14 (0.8)	2 (0.1)
	Stone	2 (0.1)	6 (0.3)
	Razor (old)	13 (0.8)	16 (0.9)
	Other	2 (0.1)	9 (0.5)
	Missing	3,207	3,894
	Not asked^1^	537	595
**For deliveries where an instrument was used: How were any surgical instruments cleaned?**^**2**^	Placed in boiling water	91 (1.7)	111 (1.7)
	Wiped with alcohol solution	36 (0.7)	22 (0.3)
	Placed over open flame	0 (0.0)	0 (0.0)
	Wiped clean	209 (3.8)	227 (3.6)
	Other	23 (0.4)	20 (0.3)
	Don’t know	309 (5.7)	368 (5.8)
**What was used to dress the umbilical cord?**	Cow dung	1 (0.0)	0 (0.0)
	Turmeric	1,363 (28.3)	1,172 (20.9)
	Tobacco	4 (0.1)	3 (0.1)
	Antiseptic	1,694 (35.2)	2,346 (41.8)
	Ash	2 (0.0)	0 (0.0)
	Nothing	1,373 (28.5)	1,658 (29.5)
	Other	376 (7.8)	440 (7.8)
	Missing	127	141
	Not asked^1^	533	590
**Delivery procedures: Was a disposable kit used?**	Yes	3,744 (78.2)	4,407 (80.5)
	Missing	154	288
	Not asked^1^	530	589
**Delivery procedures: Was the baby wiped and wrapped?**	Yes	2,892 (62.4)	3,765 (69.8)
	Missing	311	370
	Not asked^1^	531	589
**Was the baby weighed at birth?**	Yes	3,667 (75.9)	4,595 (80.9)
	Missing	111	85
	Not asked^1^	533	589
**Was the baby breastfed?**	Yes	4,711 (95.5)	5,668 (96.8)
	Missing	8	5
	Not asked^1^	531	591
**For all those breastfed: When was breast feeding initiated?**	Immediately or within half an hour after birth	6 (0.2)	7 (0.1)
	Hours after birth	2,761 (58.6)	3,509 (63.1)
	Days after birth	1,941 (41.2)	2,049 (36.8)
	Missing	3	3

^1^Not asked because it was usually felt insensitive to ask these questions of women whose deliveries did not lead to a live birth.

^2^Categories not mutually exclusive.

ANM, auxiliary nurse midwife; LHV, lady health visitor; NPHC, non-public health centre.

**Table 7:** Satisfaction with care (based on pregnancies with estimated delivery date on or after the assessment start date).

Item	Answer	Control arm (*N =* 5,474)	Intervention arm (*N =* 6,351)
**Reasons for not being fully satisfied with healthcare in pregnancy**^**1**^^**,**^^**2**^	Cost too much	1,603 (32.5)	1,625 (28.2)
	Facility not open	405 (8.2)	349 (6.1)
	Too far/transportation	1,696 (34.3)	1,887 (32.8)
	Don’t trust facility	558 (11.3)	699 (12.1)
	No female provider at facility	11 (0.2)	12 (0.2)
	Husband/family did not allow the full receipt of care	11 (0.2)	9 (0.2)
	Not necessary	55 (1.1)	27 (0.5)
	Not customary	4 (0.1)	12 (0.2)
	Other	3,791 (76.8)	4,366 (75.8)
	Not relevant, as fully satisfied	38 (0.8)	191 (3.3)
	Missing	8	5
	Not asked^3^	528	587
**Women’s rating of healthcare at delivery**	Very bad	4 (0.1)	14 (0.3)
	Bad	779 (15.8)	725 (14.7)
	Good	4,113 (83.4)	4,667 (94.6)
	Very good	35 (0.7)	342 (6.9)
	Missing	12	15
	Not asked^3^	531	588
**Reasons for not being fully satisfied with healthcare at delivery**^**1**^^**,**^^**2**^	Cost too much	1,833 (37.1)	1,416 (24.6)
	Facility not open	502 (10.2)	476 (8.3)
	Too far/transportation	1,557 (31.5)	1,751 (30.4)
	Don’t trust facility/poor quality service	915 (18.5)	1,048 (18.2)
	No female provider at facility	72 (1.5)	80 (1.4)
	Husband/family did not allow the full receipt of care	13 (0.3)	15 (0.3)
	Not necessary	38 (0.8)	37 (0.6)
	Not customary	3 (0.1)	7 (0.1)
	Other	3,729 (75.5)	4,240 (73.6)
	Not relevant, as fully satisfied	45 (0.9)	359 (6.2)
	Missing	11	11
	Not asked^3^	530	587
**Women’s rating of healthcare 28 days post-delivery**	Very bad	5 (0.1)	17 (0.3)
	Bad	508 (10.3)	473 (8.2)
	Good	4,383 (88.9)	5,107 (88.8)
	Very good	35 (0.7)	151 (2.6)
	Missing	12	15
	Not asked^3^	531	588
**Reasons for not being fully satisfied with healthcare 28 days post-delivery**^**1**^^**,**^^**2**^	Cost too much	938 (19.0)	1,049 (18.2)
	Facility not open	385 (7.8)	339 (5.9)
	Too far/transportation	1,226 (24.8)	1,509 (26.2)
	Don’t trust facility/poor quality service	505 (10.2)	620 (10.8)
	No female provider at facility	41 (0.8)	46 (0.8)
	Husband/family did not allow the full receipt of care	10 (0.2)	6 (0.1)
	Not necessary	172 (3.5)	162 (2.8)
	Not customary	3 (0.1)	6 (0.1)
	Other	3,955 (80.1)	4,588 (79.7)
	Not relevant, as fully satisfied	57 (1.2)	173 (3.0)
	Missing	11	11
	Not asked^3^	530	587

^1^Categories not mutually exclusive.

^2^Answers were free response (not multiple choice).

^3^Not asked because it was usually felt insensitive to ask these questions of women whose deliveries did not lead to a live birth.

### Economic evaluation

The total cost of the CHAMPION interventions (US$1,084,955) was divided into spending on the clinical programme (63.4%—mainly financing mobile clinics, midwives, and emergency services, with around a quarter for the NPHCs), spending on the community mobilisation programme (mainly via PDGs; 20.2%), and administration costs (16.4%). Wages were 60% of total costs (Table 8). The total costs translate into a cost of US$11,769 per life saved (95% CI $7,115 to $34,653).

**Table 8:** Costs by category (calculated in May 2011 US dollars).

Category	Cost (US dollars)	Percent of total cost
**Clinical programme**	**687,377**	**63.4**
Coordinators, supervisors, and consultants	123,647	11.4
Midwives, field staff, and other personnel	159,317	14.7
Vehicles and fuel: ambulances and mobile clinics	155,165	14.3
Staff transport and other miscellaneous costs	20,220	1.9
Materials	44,021	4.1
Referral services	178,146	16.4
Public health centre refurbishments	6,183	0.6
Other	675	0.1
**PDGs and other community mobilisation**	**219,209**	**20.2**
Supervisors and consultants	4,446	0.4
Village health workers	205,094	18.9
Events and campaigns	4,592	0.4
Other	5,078	0.5
**Administration**	**178,370**	**16.4**
Personnel	147,311	13.6
Materials	11,257	1.0
Capital costs	2,847	0.3
Other	16,954	1.6
**Total cost**	**1,084,955**	**100.0**

PDG, participatory discussion group.

Though there were substantial distances between intervention villages due to the trial design, fuel costs represented only 9% of total costs. Since all costs were local, exchange rate movements have a one-to-one impact on cost-effectiveness measures when converted to dollars.

Life expectancy at birth for those living in rural AP was reported as 68.3 years [28] and, discounted at 3%, implies 28.8 LYS per child saved, giving a cost of US$409 (95% CI $247 to $1,203) per LYS (Table 9).

**Table 9:** Indicators of cost-effectiveness.

Indicator	Value	95% Confidence Interval
Total cost	$1,084,955	
Number of deaths averted	92.19	31.31 to 152.49
Life years saved (64.3 years life expectancy, 3% discount rate, 28.8 years per life saved)	2,655	901 to 4,391
Cost per life saved	$11,769	7,115 to 34,653
Cost per life year saved (3% annual discount rate)	$409	247 to 1,203
Annual cost per population	$2.41	
Annual cost per eligible-aged woman	$14.63	
Cost per live birth	$102.14	

Costs given in May 2011 US dollars.

## Discussion

The CHAMPION trial showed that a package of interventions addressing health knowledge and health seeking behaviour, buttressing existing health services, and contracting out important areas of maternal and child healthcare led to almost the hypothesised 25% reduction in neonatal mortality in small villages in an Indian state with high mortality rates. This was in spite of large secular reductions (over 30%) in neonatal mortality in rural India over recent decades [40] (although the rate was still higher than assumed in the initial power calculations). Possible mechanisms for the excess reduction in the intervention arm may relate to the fact that women became more knowledgeable about risk symptoms for neonatal problems; had drawn up a specific birth plan; were proactively monitored, with appropriate referral and other actions; had the support of trained personnel available at delivery; had standardised care at the NPHCs; had antiseptic care for the umbilical cord; and initiated breastfeeding sooner than those in the control arm, However, the intervention was a ‘package’, and, while we could postulate particular causes for the large reduction, it is not possible to completely disentangle these in post hoc analyses. The benefits of the CHAMPION interventions were conservatively assumed to be limited to the 30 months for which statistical analysis of mortality was conducted. However, some of the intended benefits of this project are in terms of behaviour change and knowledge, which may have longer-term effects.

There were no clear effects of the intervention on maternal mortality, but the number of deaths meeting the WHO definition of deaths due to maternal causes over the period of the trial was low, and the confidence interval was wide. The rates were compatible with recent figures from AP [27].

The strengths of the study include its cluster RCT design, its size, and its focus on contracting out high-quality services. For instance, a team of doctors from the intervention team reviewed the quality of the care provided by NPHCs using stringent criteria before funds were released.

The potential for bias was reduced by separation of the research and intervention teams. They were housed in separate offices, did not train together or work together, were explicitly urged against communicating except when absolutely necessary, and had entirely separate reporting structures.

Villages in the control arm also received an active intervention (for primary-school-age children [31]) to reduce potential disappointment and increase the likelihood of receiving equally good quality data in both trial arms.

The 3-level consent process was agreed to by the relevant research ethics committees in 2007, and would also meet recent guidelines [41] for cluster RCTs.

A limitation of the study is that it could not be masked post-randomisation. It seems unlikely, however, that this led to ascertainment bias in the mortality outcomes given that these are ‘hard’ endpoints, and because of the training given to the data collectors and the standardised approaches used in the VAs. Also, if there was a bias in favour of the intervention villages, this might be expected to apply to both mortality outcomes, not only neonatal mortality.

A second limitation is that fetal losses were not divided into stillbirths and miscarriages based on gestational age, both because gestational age was not reliably reported and because of potential biases, as women in the intervention arm may have reported pregnancies earlier than control women. However, rigorous training was conducted to help data collectors to distinguish between neonatal and fetal losses.

Proportionately more women were recruited in the trial post-randomisation in the intervention arm, which might reflect women wishing to access the intervention package. While it seems implausible that women would marry into a village purely for that reason, the implications were nevertheless explored by an analysis of the interaction of arm with timing of enrolment, which was found to be significant.

It is possible that the effects of the intervention could have been diluted by other changes during the trial period, as the government introduced other maternal and child health programmes, including incentives to encourage institutional deliveries and care. Facility-based newborn care increased with the introduction of 2 government initiatives: the Janani Suraksha Yojna (JSY), a cash transfer incentive scheme for promoting institutional deliveries introduced in 2005, and the Janani Shishu Suraksha Karyakram, a free maternity and newborn programme in all government healthcare institutions introduced in 2011 under the National Rural Health Mission [42]. One of the key elements of the National Rural Health Mission is the involvement of the ASHAs. The CHAMPION trial cooperated with ASHAs in our villages when possible, but their presence and authority on maternal and child health was not fully established in most of our villages until well after the end of the trial. During the trial and afterwards, due partly to lack of manpower, blood storage facilities, and referral linkages, the public sector was often not in a position to provide emergency obstetric care services [43–47]; major problems were transportation distances and difficult terrains. The services offered under the CHAMPION trial improved access to maternal and newborn healthcare services that were not easily available to the marginalised populations living in difficult-to-reach villages.

As the aim of the CHAMPION trial interventions was not to run a parallel system, there was constant dialogue between personnel from the public health system and the trial intervention team to ensure that care of pregnant women and their babies did not fall between the gaps. Women delivering in private health facilities did not normally come under the ambit of the JSY scheme. However, those registered in the CHAMPION intervention villages could choose either the nearest government health facility or the NPHCs; many women with high-risk pregnancies opted for the NPHC, and an agreement was reached with government officials that women who gave birth at the NPHCs would be eligible to receive the JSY. Although not asked specifically about JSY, 85% of respondents in both the intervention and control arms said they expected to receive a subsidy.

The CHAMPION interventions may have reduced the demands on public health services, although the trial did not collect adequate usage and cost data to estimate the scale of this. Compared to similar interventions in India [48,49], the CHAMPION trial focused more on outreach services in the community, encouraging the majority of women to continue using existing care.

Recent reviews [50,51] seem to support the conclusions from the CHAMPION trial, in particular ‘the value of integrating maternal and newborn care in community settings through a range of interventions, which can be packaged effectively for delivery through a range of community health workers and health promotion groups’ [50].

In terms of the economic evaluation, investigators in a systematic review of cluster RCTs of women’s group interventions in India and Africa [52] found that the incremental cost per life saved ranged from $2,770 to $22,971 (2011 US dollars). In several cases, their costs were lower than the costs reported here; however, these other trials did not provide additional clinical services nor train VHWs. The CHAMPION trial was not able to determine the relative importance of the clinical services compared to the discussion groups offered. It is possible that there may be returns to scale that could further reduce the CHAMPION intervention costs, but total costs are most sensitive to wages (60% of total costs), and a scale-up of this project would not save on personnel and would not therefore lower costs significantly.

A cost-effectiveness ratio per LYS of double the local income per capita can be considered a threshold for public support [53]. This equates to US$3,180 in India (income per capita in India being US$1,590 in 2011). The cost per LYS of the CHAMPION intervention is approximately one-tenth of this threshold, suggesting that the intervention can be strongly justified on economic grounds. Given the successful results of the CHAMPION and STRIPES trials, we are continuing the interventions for a further 5 years, and scaling up the intervention to include all control villages.

In terms of generalisability, it is likely that the CHAMPION intervention can be strongly justified in much of rural India with disadvantaged populations (but with the work of the CHAMPION trial VHWs now being done by the ASHAs), and is of potential use in rural settings in other countries with similar socioeconomic and cultural patterns, similar neonatal mortality profiles, and the existence of some private health provision. Ongoing changes in maternal and child health programmes, both during the period of the trial and subsequently, make it imperative that a similar intervention that establishes ties between the community and health facilities is tested in different settings. We are in the process of designing a similar trial in a different region of India.

## Supporting information

S1 Data Directory. (XLS)

S2 Data Flow chart for women. (CSV)

S3 Data Flow chart for pregnancies. (CSV)

S4 Data Flow chart for children. (CSV)

S5 Data Comparability of villages. (CSV)

S6 Data Comparability of women. (CSV)

S7 Data Comparability of multiple births. (CSV)

S8 Data Comparability by sex. (CSV)

S9 Data Neonatal deaths. (CSV)

S10 Data Cause of neonatal deaths. (CSV)

S11 Data Neonatal deaths by stratum. (CSV)

S12 Data Maternal mortality. (CSV)

S13 Data Risk symptoms for neonates. (CSV)

S14 Data Care experience. (CSV)

S15 Data Satisfaction with care. (CSV)

S1 Fig Antenatal card: Front. (JPG)

S2 Fig Antenatal card: Rear. (JPG)

S1 Text Risk assessment criteria. (DOCX)

S2 Text CONSORT checklist. (DOCX)

## Abbreviations

ANM: auxiliary nurse midwife
AP: Andhra Pradesh
ASHA: Accredited Social Health Activist
DMC: data monitoring committee
ECL: Ensuring Children Learn
EDD: expected delivery date
FDHS: fixed day health service
ICC: intra-cluster correlation coefficient
JSY: Janani Suraksha Yojna
LSHTM: London School of Hygiene & Tropical Medicine
LYS: life year saved
NMR: neonatal mortality rate
NPHC: non-public health centre
PDG: participatory discussion group
RCT: randomised controlled trial
TSC: trial steering committee
VA: verbal autopsy
VHW: village health worker
