# Supplementary figures and images for: Community health promotion and medical provision for neonatal health—CHAMPION cluster randomised trial in Nagarkurnool district, Telangana (formerly Andhra Pradesh), India
Source: PLoS Med. 2017 Jul 5;14(7):e1002324. doi: 10.1371/journal.pmed.1002324 (PMC5497957; doi:10.1371/journal.pmed.1002324)

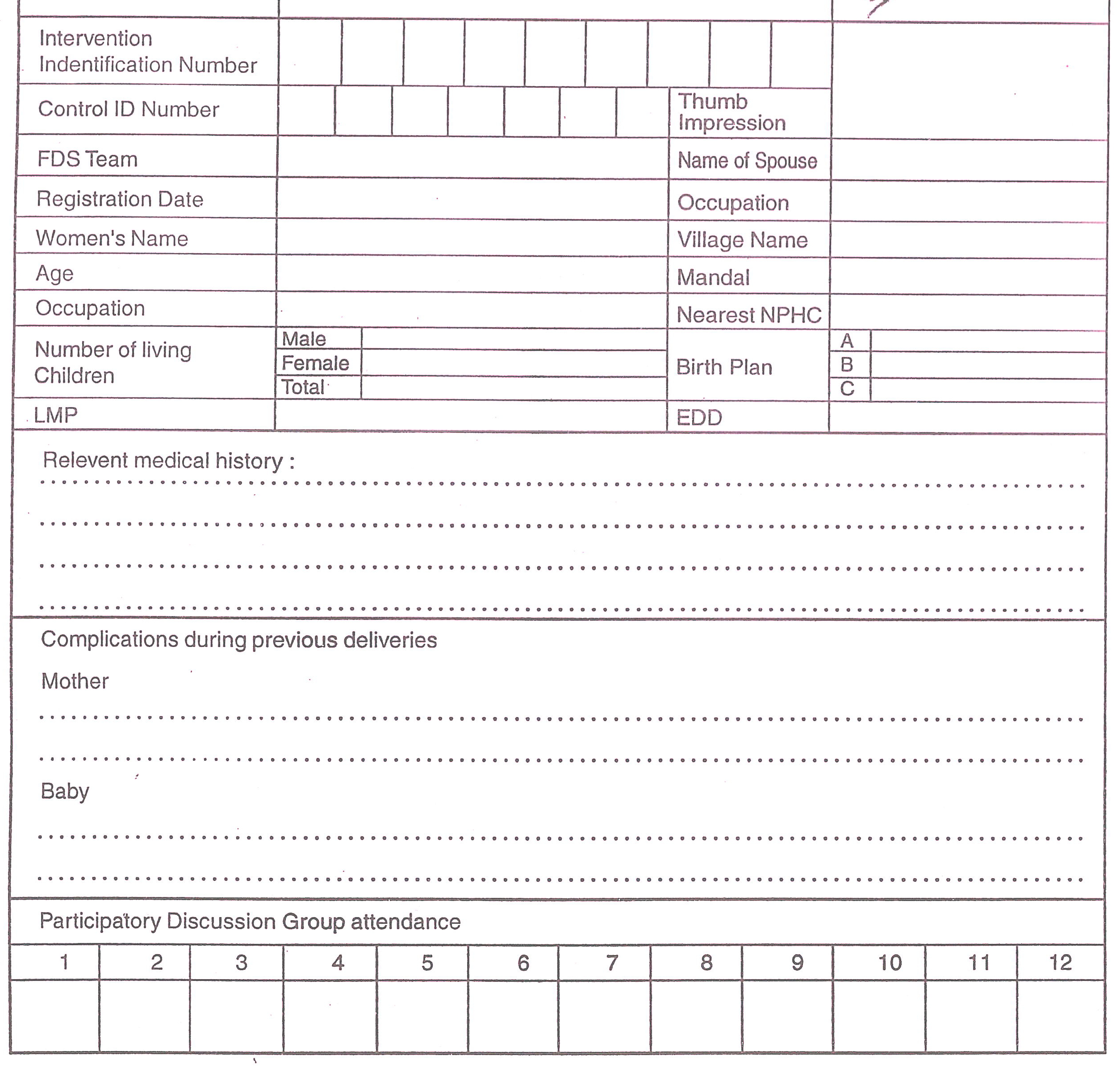

Supplement: S1 Fig — (JPG) [file pmed.1002324.s016.JPG]

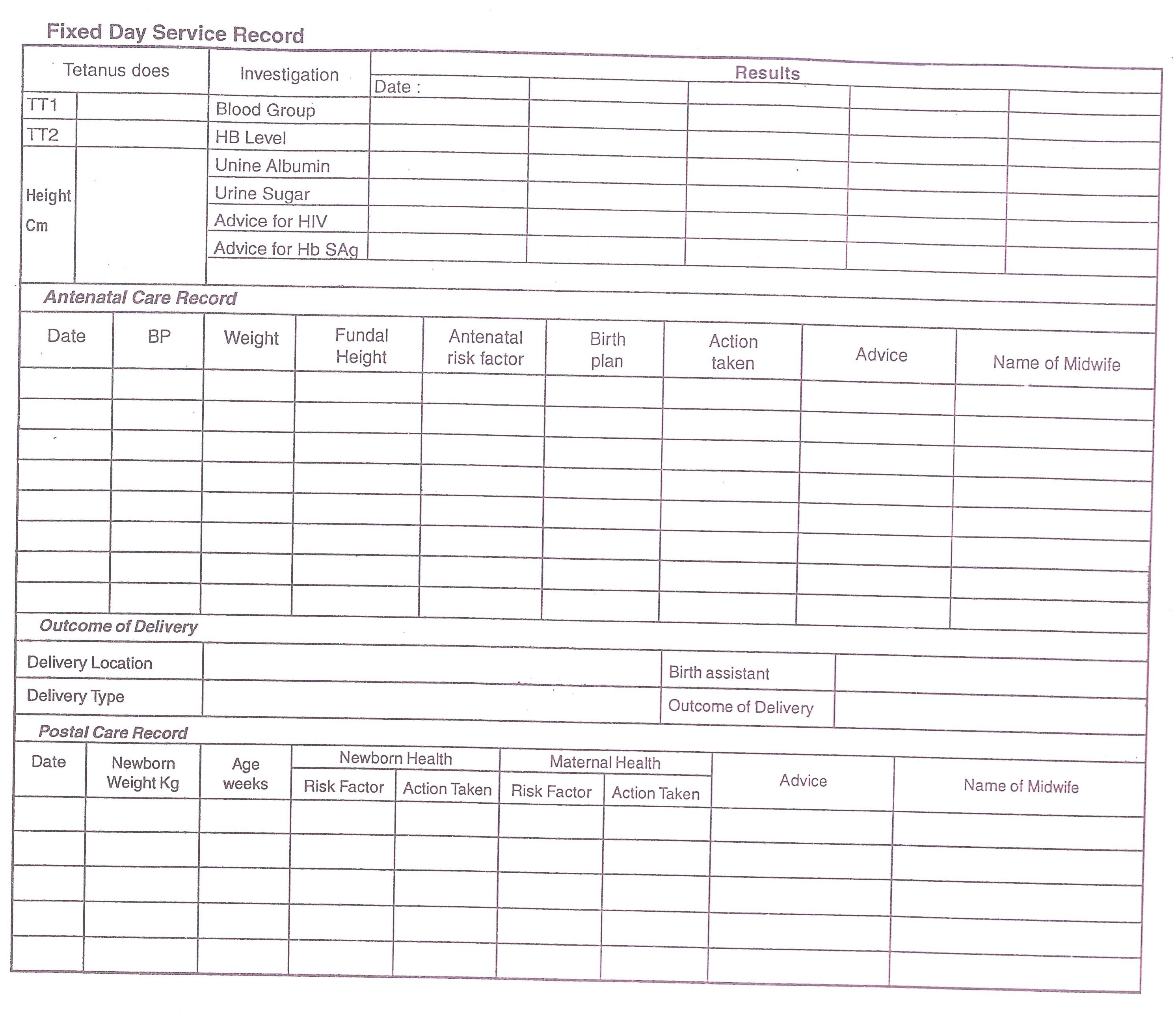

Supplement: S2 Fig — (JPG) [file pmed.1002324.s017.JPG]
