# Supplementary material for: Community health promotion and medical provision for neonatal health—CHAMPION cluster randomised trial in Nagarkurnool district, Telangana (formerly Andhra Pradesh), India
Source: PLoS Med. 2017 Jul 5;14(7):e1002324. doi: 10.1371/journal.pmed.1002324 (PMC5497957; doi:10.1371/journal.pmed.1002324)
Supplement: S1 Text — (DOCX) [file pmed.1002324.s018.docx]

| **Code** | **Risk indicator** | **Action taken** |
| --- | --- | --- |
| A1 | Severe anaemia (below 9gms) during 3^rd^ trimester | Referred to tertiary care |
| A2 | Antepartum haemorrhage, current (see plan for recent) | Emergency evacuation to tertiary care |
| A3 | Young age (below 16), if more than one hour distance from NPHC. | Counseling, referred to VHW for additional observation and monitoring |
| A4 | Elderly prime (above 35 years) | Counseling, referred to VHW for additional observation and monitoring |
| A5 | Low weight (below 40 kgs) | Counseling, referred to VHW for additional observation and monitoring |
| A6 | Moderate anaemia (9-12gms) | Referred to NPHC for treatment |
| A7 | Previous operated delivery (LSCS) | No action taken |
| A8 | Bad obstetric history (BOH) (repeated abortions, still births) | Counseling, referred to VHW for additional observation and monitoring |
| A9 | HSV infection, active or dormant | Counseling, referred to VHW for additional observation and monitoring |
| A10 | Frequent recurrent UTI (possibly leading to growth retardation, preterm delivery) | Referred to NPHC for treatment |
| A11 | History of high blood pressure | Counseling, referred to VHW for additional observation and monitoring |
| A12 | History of fits or convulsion | Referred to NPHC for treatment |
| A13 | Diabetes confirmed | Referred to NPHC for treatment |
| A14 | Complex presentations (all) | Counseling, referred to VHW for additional observation and monitoring |
| A15 | Multiple pregnancy (twins etc) | Counseling, referred to VHW for additional observation and monitoring |
| A16 | Ruptured membranes and no pains, not progressing labour  Premature rupture of membranes (PROM) | Emergency evacuation to NPHC |
| A17 | Skeletal abnormality – e.g. Pelvic or spinal | Counseling, referred to VHW for additional observation and monitoring |
| A18 | Multi Para (more than five deliveries) | Counseling, referred to VHW for additional observation and monitoring |
| A19 | Short height (below 5 ft) | Counseling, referred to VHW for additional observation and monitoring |
| A20 | Any other reason (Please specify with action taken) |  |

**Risk assessment criteria and actions to be taken**
